# The role of innate immune cells in the tumor microenvironment and research progress in anti-tumor therapy

**DOI:** 10.3389/fimmu.2022.1039260

**Published:** 2023-01-19

**Authors:** Chenglin Lu, Ying Liu, Nasra Mohamoud Ali, Bin Zhang, Xiaonan Cui

**Affiliations:** ^1^ Department of Oncology, The First Affiliated Hospital of Dalian Medical University, Dalian, China; ^2^ Department of Oncology, Affiliated Zhongshan Hospital of Dalian University, Dalian, China

**Keywords:** tumor microenvironment, immune cell, innate immune cells, anti-tumor treatment, immunotherapy

## Abstract

Innate immune cells in the tumor microenvironment (TME) mainly include macrophages, neutrophils, natural killer cells, dendritic cells and bone marrow derived suppressor cells. They play an anti-tumor or pro-tumor role by secreting various cytokines, chemokines and other factors, and determine the occurrence and development of tumors. Comprehending the role of innate immune cells in tumorigenesis and progression can help improve therapeutic approaches targeting innate immune cells in the TME, increasing the likelihood of favorable prognosis. In this review, we discussed the cell biology of innate immune cells, their role in tumorigenesis and development, and the current status of innate immune cell-based immunotherapy, in order to provide an overview for future research lines and clinical trials.

## Introduction

The tumor microenvironment (TME) is a complex environment, mainly including tumor cells and their surrounding immune cells, tumor-related fibroblasts, vascular endothelial cells, etc. These cellular interactions enable tumor cells to evade immune surveillance, and are the cytological mechanism of tumor progression and metastasis. There is accumulating evidence that innate immune cells (macrophages, neutrophils, natural killer cells, dendritic cells and bone marrow-derived suppressor cells) and adaptive immune cells (T cells and B cells) are important components of the TME, which are involved in oncogenesis and tumor progression. In particular, innate immune cells can functionally shape their microenvironment by secreting various cytokines, chemokines, and other factors, affecting tumor survival and development. In this review, we summarized the origin and phenotype of innate immune cells in the TME, their role in tumorigenesis and development, and the immunotherapy for tumor suppression based on innate immune cells.

## Macrophages

### Origin and phenotype

In humans, peripheral blood monocytes form two major populations: CD14^hi^ CD16^lo^ and CD14^lo^ CD16^hi^. In response to chemokines and growth factors produced by stromal and tumor cells in the tumor microenvironment, peripheral blood monocytes are locally recruited and differentiate into tumor-associated macrophages (TAMs). As a kind of innate immune cells in TME, TAM can be polarized into two types, M1 and M2. “M1” is induced by lipopolysaccharide (LPS) and interferon (IFN), and plays an anti-tumor effect. While type “M2” is induced by Interleukin-4 (IL-4) or Interleukin-13 (IL-13), and has a tumor-promoting effect (1). During carcinogenesis, macrophages initially exhibit antitumor M1-like polarization to eliminate more tumor cells. However, with tumor progression, macrophages polarize towards M2-like, promoting tumor development. Studies have shown that well-differentiated TAMs are associated with poor prognosis and decreased overall survival (2).

### The role of TAMs in the process of tumor development

TAMs in the TME promote tumor progression in different ways, such as stimulating angiogenesis and lymphangiogenesis, inducing proliferation and epithelial-mesenchymal transition (EMT) of cancer cells, promoting destruction of basement membrane and remodeling of extracellular matrix (ECM), and inducing immunosuppression of immune cells with anti-tumor effects. For instance, M2-derived vascular endothelial growth factor-A (VEGF-A) contributes to the neovascularization and inflammatory cell recruitment at tumor sites in a mouse model of skin cancer (3). Reports about Merkel cell carcinoma, a highly malignant cutaneous neuroendocrine tumor, indicated that M2 expresses high levels of vascular endothelial growth factor-C (VEGF-C), which can promote lymphangiogenesis (4). Moreover, STAT3 and STAT6 cooperating with cathepsins secreted by macrophages, can disrupt the basement membrane and reshape the extracellular matrix (ECM), thereby enhancing tumor invasion and metastasis (5). In addition, macrophages can also inhibit the function of anti-tumor immune cells. For example, macrophages with the expression of PD-L1/PD-L2 and CD80/CD86, bind to PD-1 and CTLA4, leading to impairment of TCR signaling and suppression of cytotoxic functions of T cells, and promoting tumor evolution (6).

**Figure 1:**
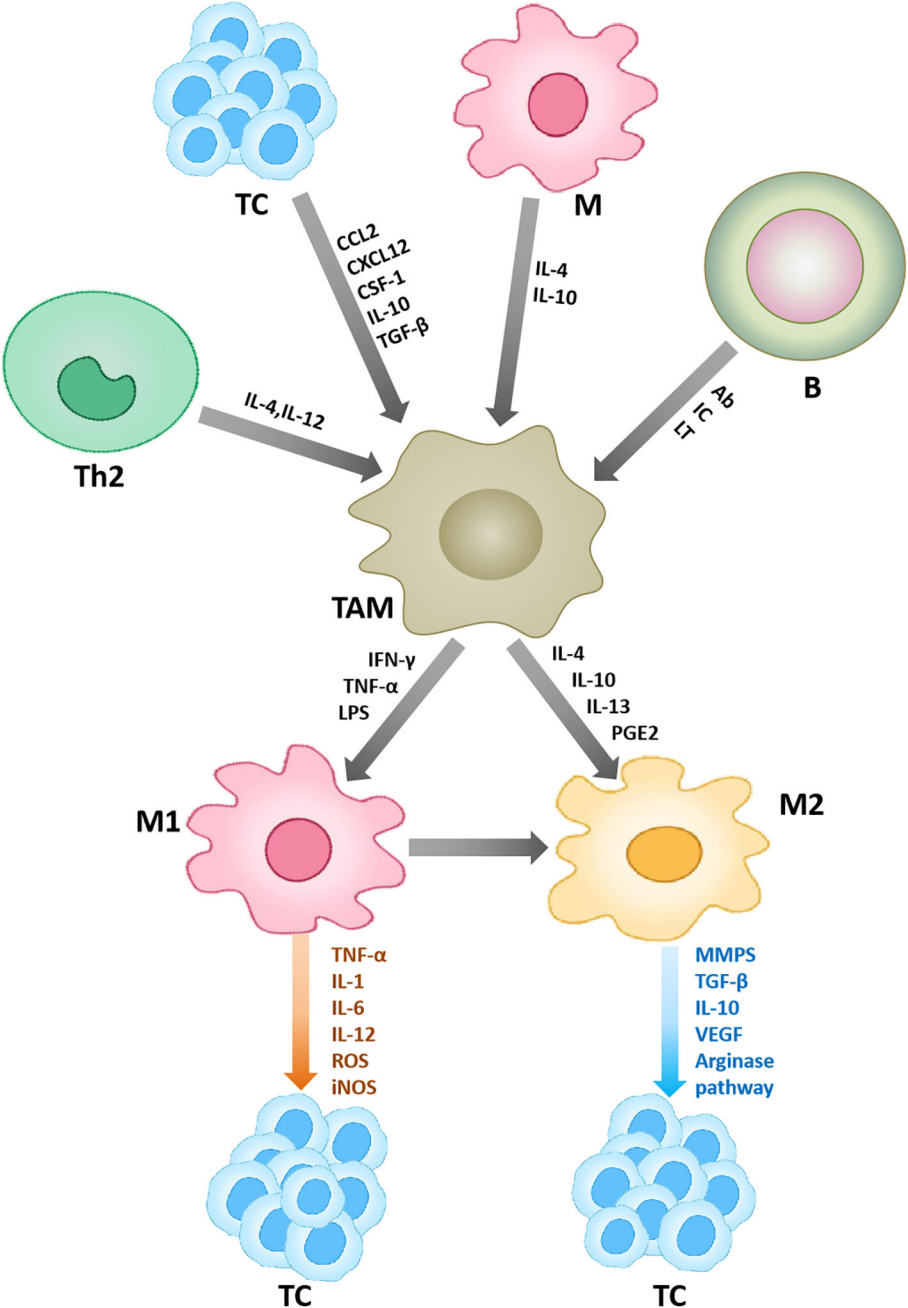
Macrophages in the tumor microenvironment. Other cells in the tumor microenvironment can affect the polarization and function of macrophages. Under the action of some cytokines, macrophages are polarized into two types, M1 and M2. M1 plays an anti-tumor role, and M2 plays a pro-tumor role. Ab, denotes antibodies; IC, denotes immune complexes; and LT, stands for leukotrienes.

Although TAMs mainly play tumorigenic roles, they can exert anti-tumor effects at times. For instance, non-classically patrolled monocytes are actively recruited to lung metastases in a CX3CR 1-dependent manner, where they eliminate tumor substances, recruit and activate natural killer (NK) cells, thereby preventing tumor cell metastasis (7). Therefore, only by comprehending the role of macrophages in the TME on tumor progression, can it be better utilized in clinical treatment (**Figure 1**).

### The role of macrophages in tumor immunotherapy

At present, the researches on macrophage immunotherapy mainly include: immune checkpoint inhibitors, monoclonal antibodies, cell adoptive therapy, small molecule inhibitors, etc, aiming to directly reduce the numbers, prevent TAMs recruitment, enhance the phagocytic killing ability or target the surface molecule of TAMs.

Studies have found that certain drugs such as emactuzumab and IMC-CS4 can inhibit tumor development by reducing the number of macrophages (8, 9). In addition, primary tumors express CCL2 and CCR2, recruiting TAM. Therefore, blocking CCL2-CCR2 could inhibit TAM recruitment. CCR2 inhibitor (PF-04136309) and Dual CCR2/CCR5 antagonists (BMS-813160) have shown efficacy in preclinical data, but the results of clinical studies are dismal (10, 11). In addition, it was found that the phagocytosis of TAM was enhanced by immune checkpoint inhibitors such as PD-1/PD-L1 inhibitors, CD47 inhibitor (Hu5F9-G4), CD40 antibody (selicrelumab), and trastuzumab, reduced tumor burden (12–15). Furthermore, small molecule inhibitors inhibit tumors by targeting molecules associated with macrophages. For example, inhibitors targeting the indoleamine 2, 3-dioxygenase (IDO) molecule on the surface of macrophages have been tested in clinical trials with promising results (16). Finally, adoptive therapy can inhibit tumor progression as well. Klichinsky et al. discovered that single infusion of human chimeric antigen receptor-macrophages (CAR-Ms) reduces tumor burden and prolongs overall survival in two xenograft mouse models of solid tumors (17).

## Neutrophils

### Origin and phenotype

Neutrophils originate from hematopoietic stem cells and can be released from the bone marrow into the circulation after maturation. They are the most abundant leukocytes in the human circulation. In response to tissue injury and infection, neutrophils overflow from the circulation into tissues under the guidance of several cytokines. They can secrete inflammatory cytokines, release neutrophil extracellular traps (NETs), and phagocytose invading pathogenic microorganisms at the site of damage tissue (18). In the context of cancer, neutrophil migration to tumor tissue is regulated by the combined effects of granulocyte colony-stimulating factor (G-CSF), interleukin-17 (IL-17), and neutrophil chemokines. Neutrophils are transformed into tumor-associated neutrophils (TANs) after migrating into tumor tissues. TANs were identified as Ly6G+CD11b+ cells (19), classifing as either an N1 (tumor suppressing) or N2 (tumor promoting) phenotype (20).

### The role of TANs in the process of tumor development

Stimulated by various factors in the TME, TANs can secrete a large number of growth factors, chemokines and cytokines to promote carcinogenesis and enhance the tumor invasion. For instance, in human pancreatic ductal adenocarcinoma (PDAC) tissues, TANs-derived transforming growth factor-beta (TGF-β) induces EMT in human lung cancer tissues through the transforming growth factor-beta (TGF-β)/Smad pathway, contributing to carcinogenesis (21, 22). In addition, in human breast cancer cell lines, neutrophils can secrete the cytokine oncostatin-M (OSM) under the induction of granulocyte-macrophage colony stimulating factor (GM-CSF), and OSM promotes vascular endothelial growth factor (VEGF) expression and increases the invasive ability of tumor cells (23, 24). On the other hand, in addition to the production of various factors, neutrophils can also release certain substances from their own stores, such as myeloperoxidase (MPO), neutrophil elastase (NE) and matrix metalloproteinases (MMPs), which affect the development of tumors. It has been reported that neutrophil-derived MPO can produce hypochlorous acid in large quantities, and induce mutations in adjacent epithelial cells by enhancing the M1dG pathway (25). Furthermore, TANs-derived MMPs, especially MMP-9, enhance tumor angiogenesis and tumor cell infiltration (26, 27).

**Figure 2:**
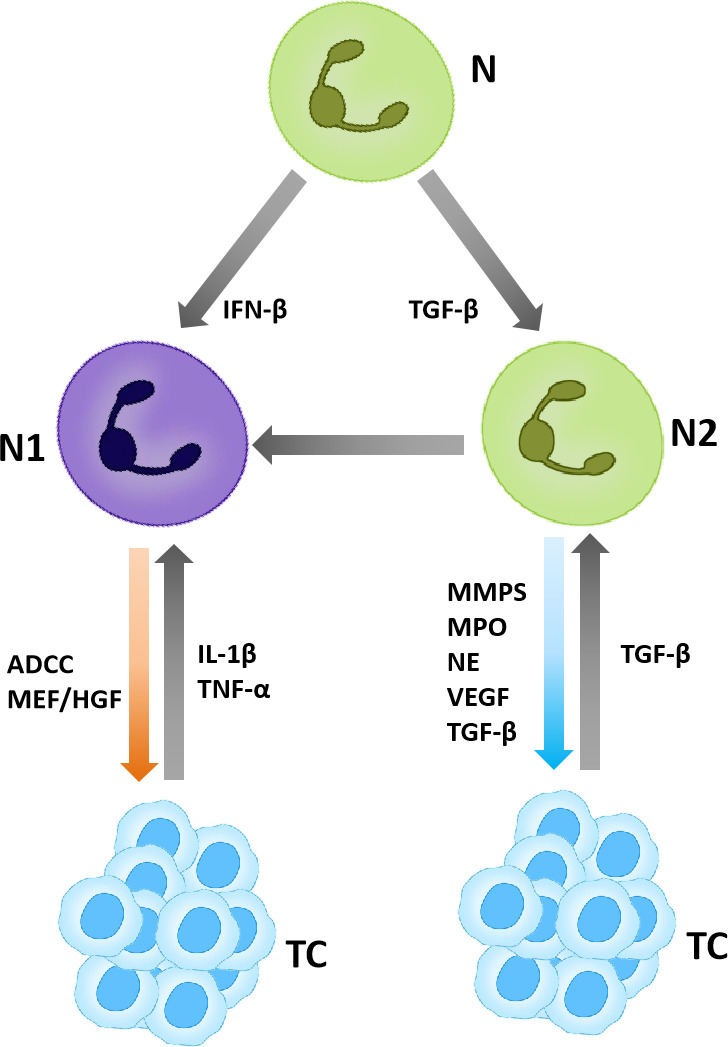
TANs in the tumor microenvironment. TANs are polarized into N1 and N2, in which N1 plays an anti-tumor role and N2 plays a pro-tumor role.

Neutrophils also play a certain tumor suppressor in some cases. It has been shown that neutrophils can exert cytotoxic effects and inhibit tumor growth by producing H_2_O_2_ in mouse breast cancer models (28). In addition, neutrophils express Fcγ receptors that mediate the elimination of cancer cells *via* the antibody-dependent cell cytotoxicity (ADCC) mechanism (29, 30). For example, in the study of lymphoma treatment in mice, it was found that the neutrophil depletion reduces the efficacy of anti-CD52 monoclonal antibody (mAb) and anti-CD20 mAb (31). In addition, natural killer (NK) cells and macrophages have also been identified as potential effector cells in anti-CD20 mAb mediated tumor regression (32–34) (**Figure 2**).

### The role of TANs in tumor immunotherapy

TANs are one of the cellular targets of immunotherapy. Current research about targeted TANs mainly focuses on reducing the number of neutrophils, inhibiting the accumulation of neutrophils at tumor sites and changing the functional phenotype of neutrophils.

This idea of depleting neutrophil numbers has been implemented in preclinical models. However, this method will reduce the circulating neutrophils, which makes the host susceptible to infection, so it is limited in clinical application (35–37). Moreover, neutrophils will migrate toward the tumor in response to chemokines, especially CXCR2, CXCR4 and G-CSF/IL-17 axes. Therefore, targeting these molecules can reduce the accumulation of TANs at tumor sites and inhibit tumor progression. Studies indicate that inhibition of CXCR2 decreases TAN accumulation, reduces lung tumorigenesis, and inhibits PDAC metastasis in mouse models (38, 39). In addition, considering the plasticity of TAN, such as tumorigenic N2-like and antitumoral N1-like phenotypes, modification of TAN phenotype might be a more desirable anticancer therapy. This hypothesis has been supported by a mathematical model that achieves the phenotypic conversion of TANs from N2 to N1 through the use of TGF-β inhibitors for antitumor purposes (40). Finally, the suppressive effects of certain drugs targeting neutrophils, such as INCB001158 (Incyte Corporation) and IPH5401 (Innate Pharma), were found to reduce tumor burden (41, 42).

## Natural killer cells

### Origin and phenotype

Natural killer (NK) cells, derived from pluripotent hematopoietic stem cells in the bone marrow and developed from lymphoid progenitor cells and NK/T cell precursors, belong to the innate lymphoid cell family and play a key role in defense against viruses and tumors. NK cells can be found both in the blood and in various lymphoid and non-lymphoid organs. Traditionally, NK cells in peripheral blood can be subdivided into two main subsets: CD56^bright^CD16^dim^ and CD56^dim^CD16^bright^. The former is considered immature and cytokine producers, while the latter shows more maturity and is the most cytotoxic (43).

### The role of NK cells in the process of tumor development

The effect of NK cells on tumors is mainly depends on the receptors expressed on their surface. These receptors fall into two main categories: activating receptors (such as NKRs) and inhibitory receptors (such as KIRs), which control the activation of NK. In healthy cells, there is no or low expression of ligands for NK cell activating receptors, and NK cells are inhibited (44). In contrast, tumorigenic or virus-infected cells express high levels of ligands for NK cell activating receptors, and NK cells are activated (45).

**Figure 3:**
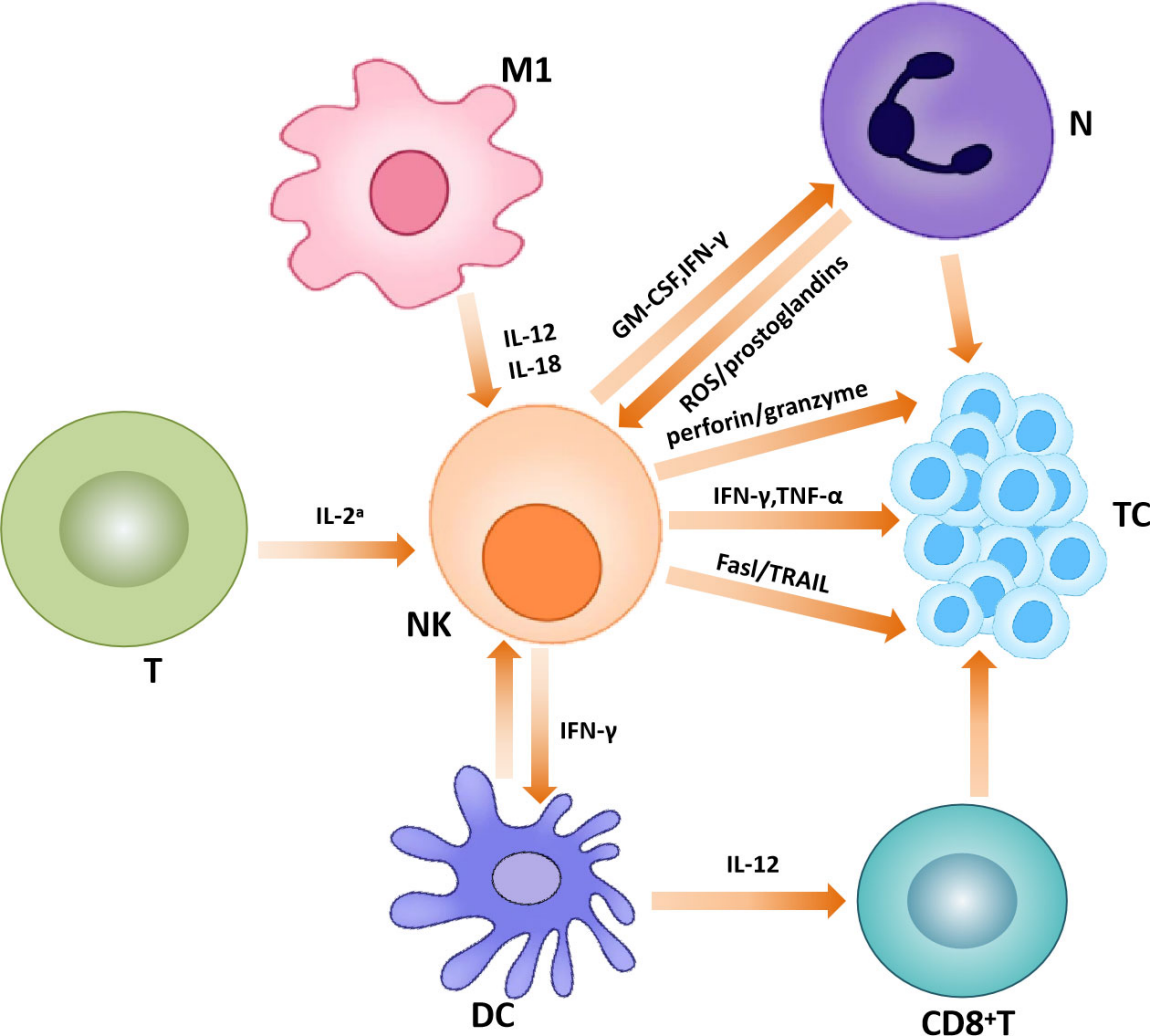
Natural killer cells in the tumor microenvironment. Natural killer cells interact with other immune cells to exert anti-tumor effects.

In the TME, activated NK cells play a killing role on tumor cells, mainly through two mechanisms: direct killing effect (natural cytotoxicity) and ADCC pathway. In the former, NK cells can recognize activating ligands on the surface of tumor cells. In the ADCC pathway, NK cell receptors bind the Fc portion of IgG antibodies to antigenic molecules on the surface of target cells. Both of these approaches lead to phosphorylation of immunoreceptor tyrosine activation moieties (ITAMs) in the cytoplasm domain of receptors, which initiates NK cell activation. Activated NK cells can kill tumor cells by directly releasing cytotoxic granules containing granzymes and perforin or by inducing death receptor-mediated apoptosis *via* the expression of Fas ligand or tumor necrosis factor-related apoptosis-inducing ligand (45, 46). In addition to the ITAM pathway, there is also the PI3K pathway associated with its killing ability (47, 48). In addition to killing tumor cells in the above two ways, NK cells secrete chemokines and cytokines to play an immunomodulatory function as well. studies indicated that NK cells can also produce chemokines, C-C-motif ligands such as CCL3, CCL4, CCL5, X-C-motif chemokine ligand 1 (XCL1) and C-X-C-motif chemokine ligand 8 (CXCL8), which attract a variety of immune cells to transformed tissues and inhibit tumor progression (49). However, in addition to playing an anti-tumor role, certain subtypes of NK cells may contribute to tumor development. For example, IL6-induced overexpression of CD39 on NK cells is associated with poor prognosis in ESCC (50). In addition, some studies have shown that the poor prognosis of some cancers such as lymphoma, breast cancer and gastric cancer is related to natural killer cells, which may be related to the limited killing ability of NK cells. The detailed mechanism needs to be further explored (51–53) (**Figure 3**).

### The role of NK cells in tumor immunotherapy

In recent years, the research on NK cell immunotherapy has flourished, and the latest progress mainly focuses on cytokine supplementation, monoclonal antibodies, modification of internal signaling pathways, adoptive transfer of NK cells and genetic engineering, etc.

Supplementation of cytokines such as IL-2, IL-15, IL-18 and IL-21 can improve the activity of NK cells and enhance their killing ability on tumors. For example, recombinant human IL-15 and its agonist ALT803 have great therapeutic efficacy in metastatic lung cancer (54, 55). ALT-803 combined with anti-PD-1 monoclonal antibody shows better therapeutic potential than ALT803 alone (56, 57). Furthermore, in one study, NK-cell dysregulation and cytotoxicity were restored in patients with mesothelial carcinoma treated with the monoclonal antibody against CTLA4, telemumab, indicating that blockade of CTLA4 prevents NK-cell dysfunction, slows tumor growth, and improves overall survival among patients with tumors (58). In addition, as an option to enhance autoimmunity, adoptive transfer of NK cells has been used for the treatment of certain types of cancers, such as medulloblastoma and ependymoma in children. In this study, medulloblastoma upregulates ligands of NK cell-activating receptors such as NKG2D and natural cytotoxic receptors (NCRs), thereby inducing NK cell-mediated cytotoxicity and medulloblastoma cell apoptosis, demonstrating the feasibility and safety of intraventricular infusion of autologous NK cells. It also provides a theoretical basis for local-regional delivery of NK cells (59). Finally, CAR-NK, which is genetically modified by chimeric antigen receptors (CARs) on immune cells, directly targets tumor-specific antigens, and has shown promising results in preclinical studies in ovarian, breast, prostate, and colon cancers (60).

## Dendritic cells

### Origin and phenotype

Dendritic cells (DCs) are typical antigen-presenting cells (APC) in the immune system, which connect the adaptive and innate immune systems to initiate and maintain T cell-mediated antitumor immune responses. DCs originate from common myeloid progenitor cells (CMPs). Driven by the expression of the transcription factor Nur77, CMPs differentiate into monocytic dendritic cells (moDCs) under inflammatory conditions (61–63). In the absence of Nur77, CMPs differentiate into common dendritic cell progenitors (CDPs), resulting in plasmacytoid dendritic cells (pDCs) and conventional dendritic cells (cDCs) (61). In addition, cDCs can be further divided into two subsets termed type I (cDC1) and type II (cDC2) conventional DCs (64). CD123, CD303, CD304, and CD45RA are expressed in pDCs. cDC1 and cDC2 express CD141, DEC205,Clec9A, XCR1 and CD1c,CD1a,CD103, respectively (65).

### The role of DCs in the process of tumor development

DCs can continuously take up antigens within the TME and sense danger signals through pattern recognition receptors (PRRs) to recognize damage-related molecular patterns (DAMPs) from malignant cells. These signals enable DCs to initiate tolerance and immunogenicity properties in a coordinated, and subset-specific manner in general (66). Therefore, in order to better understand the role of DCs in the TME, further exploration is needed on the function of DC subsets.

**Figure 4:**
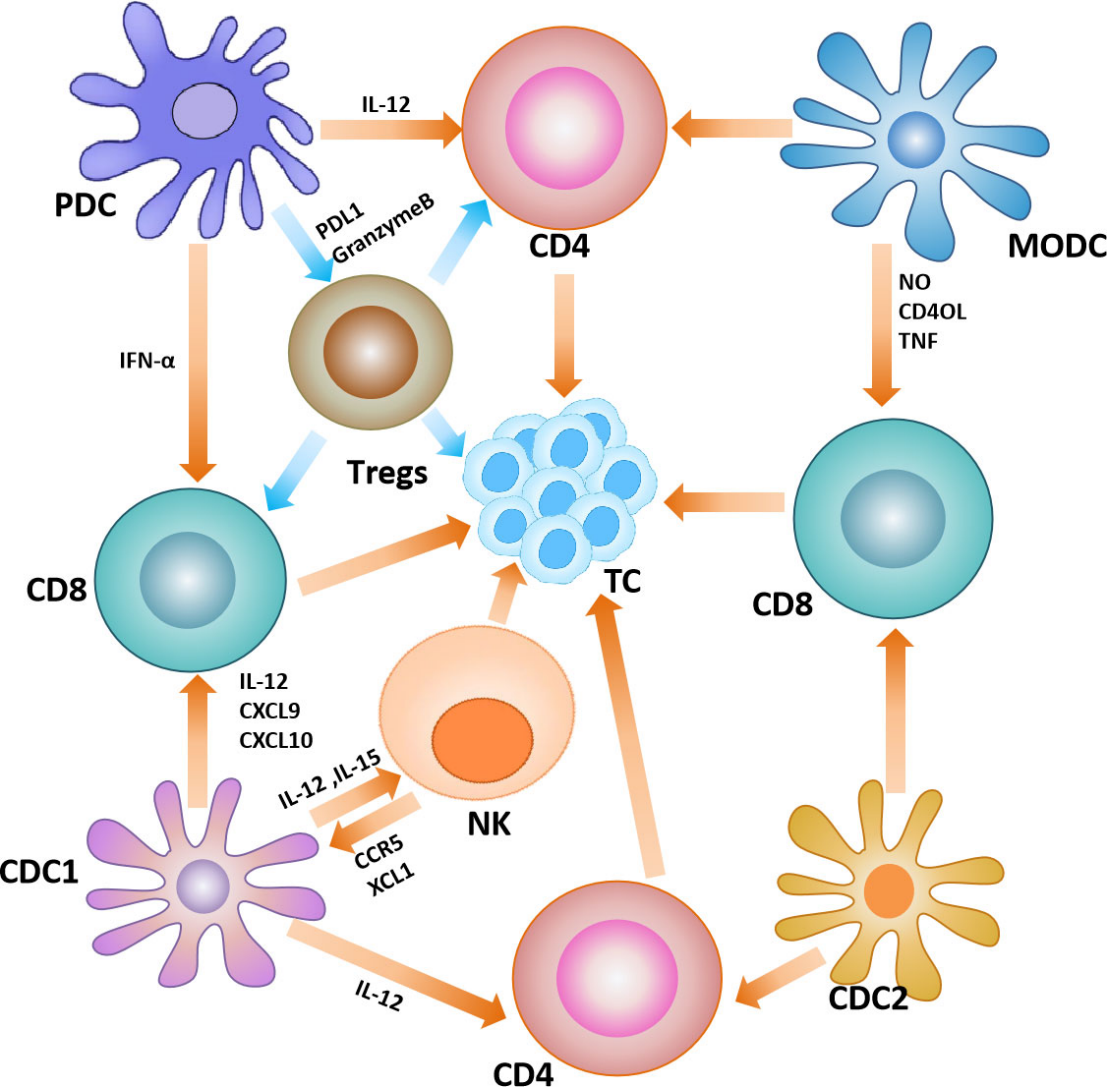
Dendritic cells in the tumor microenvironment. Interactions between dendritic cells and other immune cells in the tumor microenvironment, where yellow arrows represent anti-tumor effects and blue arrows represent pro-tumor effects.

As the most extensively studied subset of DCs, cDC1 can phagocytose exogenous antigens and other cellular debris released by tumors, and cross-present on major histocompatibility complex class I (MHC-I) to prime tumor-specific CD8+T cells (67). In addition, cDC1 can interact with natural killer (NK) cells to exert anti-tumor effects. For example, IL-12 and IL-15 produced by cDC1 can enhance the killing ability of NK cells (68). While natural killer cells produce cytokines and chemokines, such as XCL1 and CCR5, to recruit more cDC1 to TME (69). The subset of cDC2 have strong heterogeneity and functional diversity, thus their role in cancer has not been clarified. However, several researches have also indicated that cDC2 are involved in cross-presenting antigens and driving T-cell responses (70, 71). The subset of pDCs is mainly involved in antiviral responses. However, it has been found that pDCs can induce the expression of programmed cell death protein 1 ligand 1 (PD-L1) or granzyme B in malignant tumors, regulating Treg cells to drive immune tolerance (72–74). The role of MoDC in tumors is not well characterized, and may be related to cross-presentation of relevant antigens and initiation of CD8+T cell responses. In the melanoma mouse study, tumor-derived MoDCs can efficiently cross-present tumor antigens and are able to induce significant CTL proliferation (75) (**Figure 4**).

### The role of DCs in tumor immunotherapy

With accumulating evidence that DCs play a critical role in initiating antitumor immunity, there has been increasing interest in improving the status and numbers of DCs to suppress tumor progression. Currently, the following immune-related methods are mainly being applied: activation of DCs *in vivo*, blocking inhibitory signals, *in vivo* expansion, DC vaccines and so on.

Activation *in vivo* is achieved by providing exogenous activation signals, suggesting that DCs can be activated with specific agonists to shape the optimal antitumor response. Furthermore, blocking the inhibitory signals of cDCs is similar to activation *in vivo*. One of the most common examples is the application of VEGF inhibitors, which have been demonstrated to enhance DC maturation in patients (76, 77). In addition, tumors can be suppressed by increasing the number of DCs. For instance, systemic injection of the growth factor fms-like tyrosine kinase 3 ligand (Flt3L) has been shown in preclinical studies to cause expansion and activation of the cDC1 population, dramatically enhancing the clinical response to checkpoint and BRAF blockade and significantly delaying tumor growth (78). Alternatively, targeting DC by vaccines is another therapeutic option. The most common one is the whole-cell vaccines. For example, DCVax-L, a DC vaccine against glioblastoma, which has been tested in phase III clinical trials and has shown prolonged overall survival in patients (79). In addition, another example is that a neoantigen dendritic cell vaccine combined with anti-CD38 and CpG can produce anti-tumor immunity against immune checkpoint therapy resistant mouse lung cancer cell lines, which further develops DC vaccine therapy (80). DC vaccine is a promising immunotherapy method, and relevant clinical trials are ongoing (**Table 1**).

**Table 1:** Drugs and clinical therapies targeting innate immune cells.

Innate immune cell types	Mechanism	Drugs or therapeutics in immunotherapy	ClinicalTrails.gov Identifier
Macrophages	Reducing the numbers	Emactuzumab (RG7155)(8)	NCT02323191
		IMC-CS4 (9)	LY3022855
	Preventing recruitment	CCR2 inhibitor (PF-04136309)(10)	NCT01413022
		Dual CCR2/CCR5 antagonists (BMS-813160)(11)	NCT03496662, NCT03767582, NCT04123379, NCT02996110
	Enhancing the phagocytic killing ability	PD-1/PD-L1 inhibitors(14)	Marketed
		CD47 inhibitor (Hu5F9-G4)(12)	NCT04541017, NCT02953782
		CD40 antibody (Selicrelumab)(13)	NCT02304393, NCT03424005, NCT03193190, NCT03555149
		Trastuzumab(15)	Marketed
	Targeting the surface molecules	IDO inhibitors(16)	NCT02073123
	Others	CAR-M(17)	Preclinical studies
Neutrophils	Reducing the numbers	Ly6G(35, 36)	Preclinical studies
		TARIL-R2 (DS-8273a)(37)	NCT02991196
	Inhibiting the accumulation at tumor sites	Reparixin(38)	NCT02001974
		CD47-SIRPα antibody(39)	NCT02216409, NCT03717103, NCT02367196…
	Changing the functional phenotype	Galunisertib (LY2157299)(40)	NCT02452008, NCT02688712, NCT04605562
		Fresolimumab (GC1008)(40)	NCT01472731, NCT01112293
	Targeting the inhibitory effect	INCB001158 (Incyte Corporation)(41)	NCT02903914, NCT03314935
		IPH5401 (Innate Pharma)(42)	NCT03665129
NK cells	Enhancing killing ability	ALT803(54, 55)	NCT01885897, NCT01885897
		CTLA-4(58)	NCT01843374
	Others	NK cell adoptive immunotherapy(59)	NCT02271711
		CAR-NK(60)	NCT00995137, NCT01974479, NCT02742727, NCT02944162, NCT02892695, NCT02839954
DCs	Activation *in vivo*	VEGF inhibitors(73, 74)	Preclinical studies
	Expansion *in vivo*	Flt3L(75)	Preclinical studies
	DC vaccines	DCVax-L(76, 77)	NCT00045968
MDSCs	Inducing the differentiation into mature myeloid cells	ATRA(81, 82)	NCT00617409
	Reducing/Consuming the numbers	5-Fu, paclitaxel, cisplatin and gemcitabine(83–85)	Marketed
		tyrosine kinase inhibitors(86, 87)	NCT02868255
	Inhibiting the immunosuppressive activity	COX-2/PGE2 inhibitors(88)	Preclinical studies

## Myeloid-derived suppressor cells

### Origin and phenotype

Myeloid derived suppressor cells (MDSCs) are a major regulatory cell population originating from myeloid progenitor cells that can be activated by tumor-derived factors in the TME. The diversity of phenotypic characteristics of MDSC subsets is determined by the differences in soluble factors that mobilize MDSCs. MDSCs can be roughly divided into three subgroups: polymorphonuclear MDSCs (PMN-MDSCs), monocyte MDSCs (Mo-MDSCs), and early MDSCs(E-MDSCs). The phenotypes of Mo-MDSCs were CD11b+, LY6Chi, and LY6G-; PMN-MDSCs showed CD11b+, LY6Clo, and LY6G+ phenotypes; while E-MDSCs expressed CD13-, CD14-, and CD33+ phenotypes (89). Notably, both of Mo-MDSCs and PMN-MDSCs present in the TME have enhanced inhibitory phenotypes compared with MDSCs present in peripheral lymphoid organs, which is due to the increased expression of inhibitory molecules by MDSCs in the TME.

### The role of MDSCs in the process of tumor development

**Figure 5:**
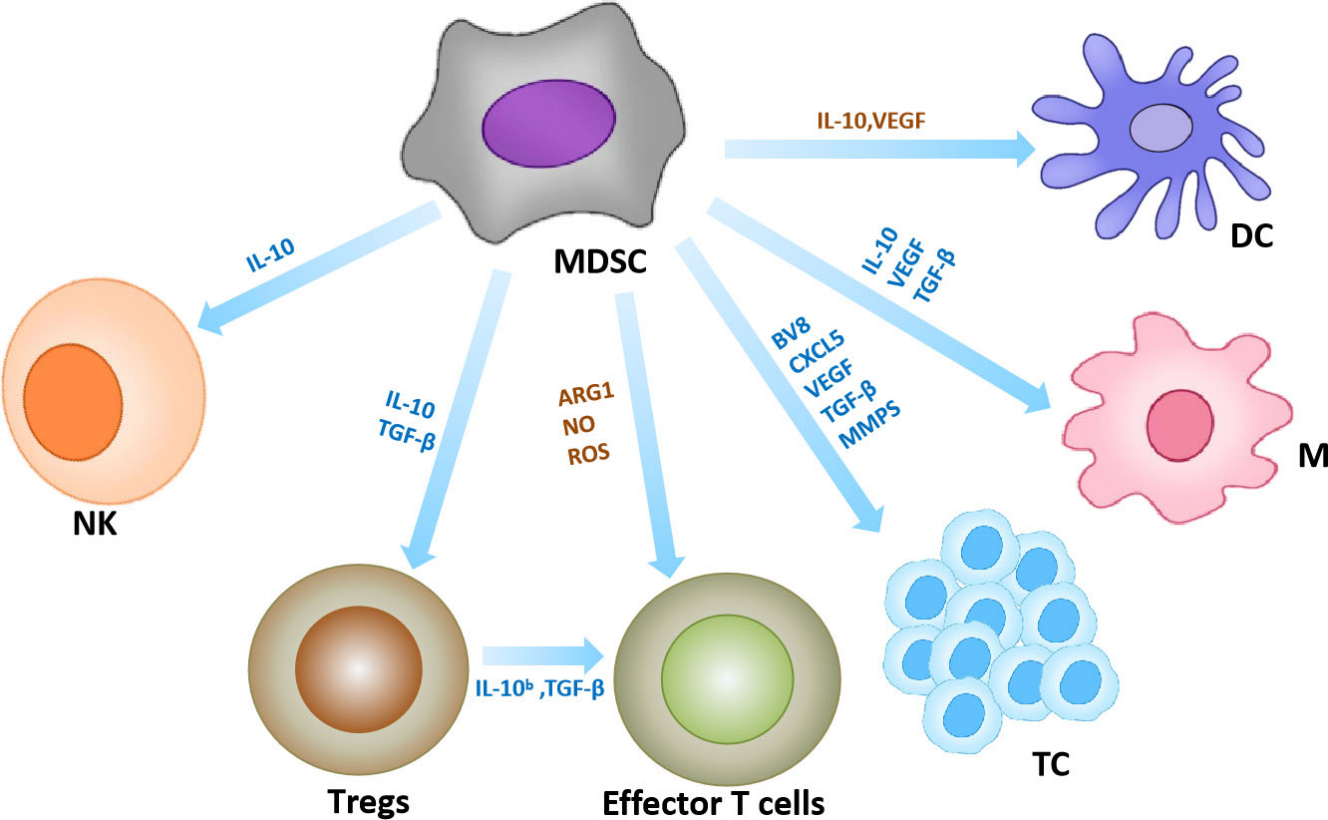
MDSCs in the tumor microenvironment. Interaction of MDSC with other immune cells. Yellow arrows represent anti-tumor effects and blue arrows represent pro-tumor effects. (95, 96).

As one of the components of the TME, MDSCs have strong immunosuppressive potential. On the one hand, MDSCs establish an immunosuppressive TME through the production of various metabolites such as ROS and nitric oxide, which inhibit the antitumor function of T cells and NK cells. For example, MDSCs induce the production of IDO and immunosuppressive cytokines that promote tumor evolution by suppressing cytotoxic T lymphocytes, DCs and NK cells, and enhancing the effects of Tregs (90). For example, MDSC inhibits effector T cell proliferation and antitumor activity by producing ROS, arginase, and NO (91–93). MDSC inhibits the cytotoxicity of natural killer (NK) cells by inhibiting IL-2-mediated activation of NK cells or direct secretion of IL-10 (81, 82, 94). MDSC can also induce Treg differentiation and promote tumor development by secreting IL-10 and TGF-β (83). On the other hand, MDSCs contribute to tumor development by creating a premetastatic niche that promotes neovascular invasion and tumor cell growth. For instance, MDSCs can secrete an important pro-angiogenic factor (Bv8) at the tumor sites to promote tumor angiogenesis (84). In addition, in a mouse melanoma model, PMN-MDSCs were found to infiltrate primary tumors and induce epithelial-mesenchymal transition (EMT) through EGF and HGF signaling pathways to promote cancer cell spread under the induction of chemokine CXCL5 (85) (**Figure 5**).

### The role of MDSCs in tumor immunotherapy

Since MDSCs have immune-suppressing effects in a variety of cancers, altering these cellular inhibitory effects is considered a promising approach for cancer treatment. Several strategies are being developed that can be broadly classified into four categories: inducing the differentiation of MDSCs into mature myeloid cells; reducing/consuming the number of MDSCs; blocking the development of MDSCs; inhibiting the immunosuppressive activity of MDSCs.

All-trans retinoic acid (ATRA) has been identified as a drug that induces the differentiation of MDSCs into mature myeloid cells. ATRA can bind to retinoic acid receptors and block signal transduction, leading to the differentiation and maturation of MDSCs. For example, in patients with metastatic renal cancer, ATRA combined with IL-2 resulted in significantly lower MDSCs and improved immune responses compared with untreated patients (86, 97). In addition, several chemotherapeutic agents such as 5-fluorouracil (5FU), paclitaxel, cisplatin and gemcitabine can deplete MDSCs and enhance the antitumor immune activity of T cells (87, 88, 98). Alternatively, it has also been reported that tyrosine kinase inhibitors reduce the number of MDSCs in renal cell carcinoma (RCC) patients and hepatocellular carcinoma (HCC) by blocking VEGF, c-KIT, and STAT-3 (99–101). Finally, inhibiting the immunosuppressive activity of MDSCs. For instance, cyclooxygenase 2 (COX-2)/prostaglandin E2 (PGE2) axis signaling antagonism has effectively reduced MDSCs recruitment and differentiation, and inhibited the production of MDSCs related inhibitors such as arginase 1 and ROS, thereby controlling tumor progression (102).

## Conclusion and prospects

Innate immune cells in TME play an important role in the occurrence and development of tumors, and even show dual effects of tumor promotion and tumor inhibition under different microenvironments and stimulators. The tumor-promoting effect shows that they can increase the stemness of tumor cells, promote angiogenesis, induce drug resistance, inhibit immunity and promote tumor growth through phenotypic changes and secretion of certain substances. Each link may become the target for clinical treatment. We summarize some drugs and treatments that target innate immune cells. (**Table 1**). An in-depth comprehending on the role of innate immune cells and related molecules will not only provide important insights into the biological behavior of different types of tumors, but also provide a necessary foundation for the development of new innate immune cell-based therapies to manage and control the cancers. Although the current immunotherapy has achieved vast strides both in improving patients’ survival and their quality of life, more and more problems have emerged, such as the narrow anti-tumor spectrum, increased adverse reactions, and easy drug resistance, etc. These defects limit the practical application of immunotherapy in the clinic. Meanwhile, the complex components and heterogeneity of the TME also pose great challenges for immunotherapy. In the future, the mechanism of immune cells on tumor development should be further explored, the heterogeneity of TME should be deeply understood and studied, more accurate tumor markers should be sought, new methods of combination therapy should be explored. Thereby, formulating the more accurate and effective individualized strategies for treatment.

## Data availability statement

The original contributions presented in the study are included in the article/supplementary materials. Further inquiries can be directed to the corresponding authors.

## Author contributions

All authors listed have made a substantial, direct, and intellectual contribution to the work and approved it for publication.
